# The impact of internet pornography addiction on brain function: a functional near-infrared spectroscopy study

**DOI:** 10.3389/fnhum.2025.1477914

**Published:** 2025-04-16

**Authors:** Qicheng Shu, Shiyu Tang, Zhenhua Wu, Jiahuan Feng, Wenhao Lv, Min Huang, Fan Xu

**Affiliations:** ^1^Department of Evidence-Based Medicine and Social Medicine, School of Public Health, Chengdu Medical College, Chengdu, China; ^2^Department of Clinic Medicine, School of Clinical Medicine, Chengdu Medical College, Chengdu, China; ^3^Department of Pharmacy, School of Pharmacy, Chengdu Medical College, Chengdu, China; ^4^Department of Physiology, School of Basic Medical Sciences, Chengdu Medical College, Chengdu, China

**Keywords:** internet pornography, addiction, functional near-infrared spectroscopy (fNIRS), brain function, cognition

## Abstract

**Introduction:**

There is extensive awareness of internet pornography addiction. It not only affects the mental health of adolescents but also promotes criminal activity. However, the impact of internet pornography addiction on functional in the brain remains unclear.

**Methods:**

16 healthy college students and five college students with severe internet pornography addiction were invited to participate in the experiment and watch a pornographic video. Functional near-infrared spectroscopy (fNIRS) was used to measure the dynamic changes in hemoglobin in the brain during a 10 min session of viewing internet pornography. Participants completed the Stroop Color and Word Task (SCWT) before and after they had watched the video. Facial expressions and life signs were measured continuously during the experiment.

**Results:**

Compared with the group that frequently viewed pornographic videos, the group with low-frequency pornography viewing exhibited enhanced functional connectivity in the inferior prefrontal cortex and pars triangularis of Broca’s area in the frontal lobe, the primary somatosensory cortex in the parietal lobe, and the pre-motor and supplementary motor cortices. Moreover, the high-frequency pornography-viewing group exhibited hyperactive parasympathetic activity, more pronounced sexual arousal, and stronger functional connectivity in the dorsolateral prefrontal cortex and frontopolar area. After viewing the pornography, the high-frequency group demonstrated longer reaction times and significantly reduced accuracy while completing the Stroop Color and Word Test (SCWT) compared to the low-frequency group and also their own performance before and after viewing the pornography.

**Discussion:**

This study demonstrated the hyperactive and inhibited brain areas under the impact of pornography video addiction. The results may strengthen our understanding of neurobiology and facilitate the development of prevention policies for adolescents.

## Introduction

With the proliferation of technology and changes in the social environment, pornographic content, especially pornographic films, has become widely disseminated through the internet. There has been a rapid increase in the frequency and duration of internet pornographic use, particularly during the 2019 coronavirus (COVID-19) pandemic (Van Loo et al., 1997; Yunengsih and Setiawan, 2021). A survey in the United States revealed that 66% of teenagers had encountered internet pornography unintentionally, while 34% had deliberately sought it out (Wolak et al., 2007). A survey conducted in China showed that in recent years, there has been a rapid increase in the frequency and amount of internet pornography accessed by adolescents (Dong et al., 2020). Due to the prolonged overactivation of the rewards system, ceasing the supernormal stimulus of watching pornography for an extended period can lead to withdrawal reactions, resulting in negative emotions such as anxiety, irritability, depression, and anger; all of these can seriously affect cognitive function (Koob and Volkow, 2010). Previous studies have found that internet pornography addiction and drug addiction exhibit similar phenomena (Love et al., 2015), but the relationship between the two has not yet been clarified, and the underlying mechanisms have not yet been fully elucidated.

The sustained and intense release of dopamine while watching pornography can lead to a strong craving for and dependence on it. A functional magnetic resonance imaging (fMRI) study on internet pornography (Kühn and Gallinat, 2014) found a significant negative correlation between the volume of the right caudate gray matter and functional activity in the left putamen cue-response paradigm in individuals who had viewed pornography for an extended period of time. The functional connection between the right caudate nucleus and the left dorsolateral prefrontal cortex correlated negatively with the duration of watching pornography. The problematic Internet pornography use scale (PIPUS) is a tool for evaluating the problematic consumption of internet pornography (Chen and Jiang, 2020). It is used to measure and identify signs of dependence and related issues in an individual’s use of internet pornography. The Stroop Color Task is a psychological test used to measure cognitive interference and the ability to manage conflicting information (Williams et al., 1996). Thanks to the rapid development of non-invasive measurement technology, functional near-infrared spectroscopy (fNIRS) is an optical, non-invasive neuroimaging technique that can measure changes in the concentrations of oxyhemoglobin and deoxyhaemoglobin in specific brain regions (Scholkmann et al., 2014). It can continuously monitor the hemodynamic changes in specific brain regions while an individual performs a specific task and simultaneously calculates the average changes in hemoglobin during this period. Compared to fMRI, fNIRS offers a more compact form and is easier to operate. Its convenient operation, including a quiet measurement environment, no limitations on task performance, and low interference with other equipment, makes it particularly well-suited for investigating alterations in brain functional connectivity during the viewing of internet pornography. Subsequently, fNIRS was used to evaluate the performance of functional connectivity in participants while they viewed pornography. Previous studies have also applied fNIRS to investigate drug abuse (Qi et al., 2022). Furthermore, life signs and facial expressions were measured continuously during the viewing. The questionnaires, including PIPUS, the self-rating depression scale (SDS) (Zung, 1965), and self-rating anxiety scale (SAS) (Dunstan and Scott, 2020), were also completed accordingly. We used the above methods to investigate the mechanisms of internet pornography addiction and identify potential treatment approaches.

## Materials and methods

### Participants

Sixteen healthy college students who occasionally watched pornographic films and five college students with severe internet pornography addiction were invited to participate in the experiment. The inclusion criteria included no illness or disease and right-handedness. The exclusion criteria included red-green color blindness, non-heterosexuality, smoking, substance abuse issues, and being unhealthy. Masturbation was prohibited during the experimental period. Each participant provided written informed consent prior to starting the experiment.

### Experimental materials and procedures

**FIGURE 1:**
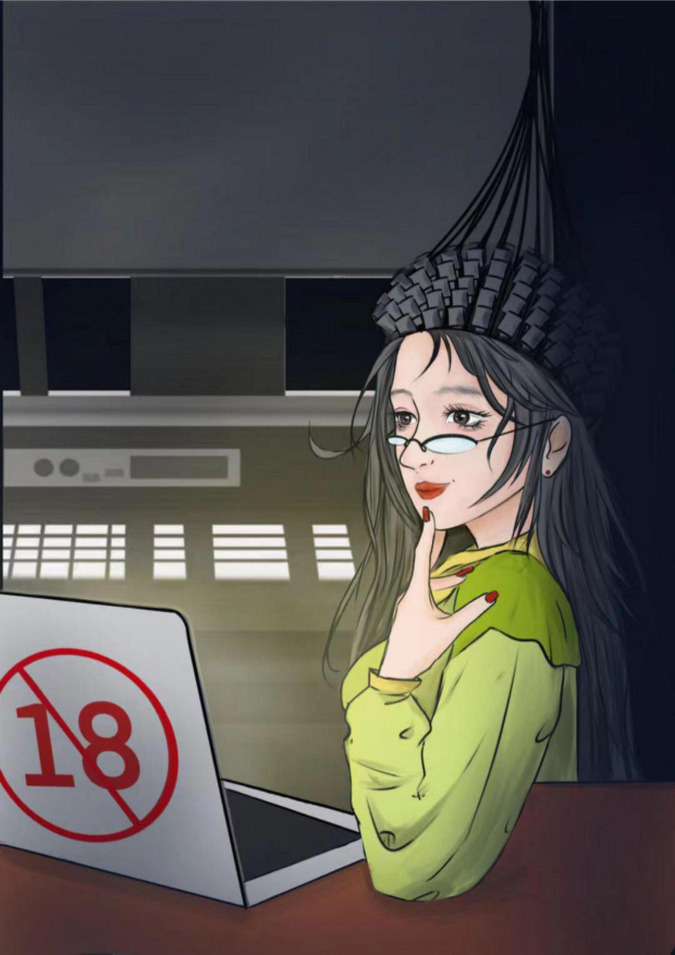
A schematic of experimental procedure.

**FIGURE 2:**
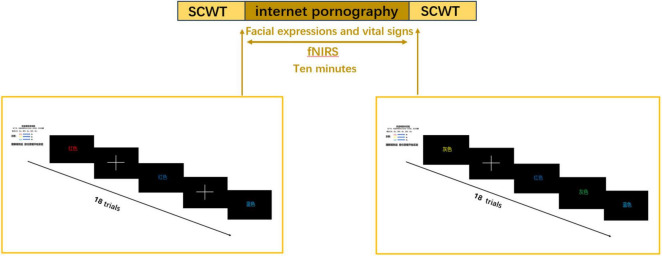
The general workflow of the study.

The SCWT was conducted using the E-Prime 3.0 software (Psychology Software Tools, Inc. United States). A 10 min internet pornographic video—selected on how often it had been viewed—was used in this test. Each participant completed the SCWT, watched the 10 min pornographic video (Figure 1), and then completed the SCWT again while changes in hemoglobin were measured with fNIRS. Their life signs and facial expressions were also recorded. The SCWT was administered 18 times per group; each trial lasted 2 s and was skipped if no response was given within the timeframe (Figure 2). Subsequently, the participants were asked to complete three survey questionnaires: PIPUS, SDS, and SAS.

### Measurement of facial expressions and vital signs

Facial expressions were measured using the Noldus Face Reader 7.0 (Noldus Information Technology, NL). The Mindray (VS-600) was used to measure the following life signs: heart rate, percutaneous arterial oxygen saturation (SpO_2_), the standard deviation of normal-to-normal interbeat intervals (SDNN), systolic blood pressure (SBP), and diastolic blood pressure (DBP) (Zhang et al., 2024).

### fNIRS data collection

**FIGURE 3:**
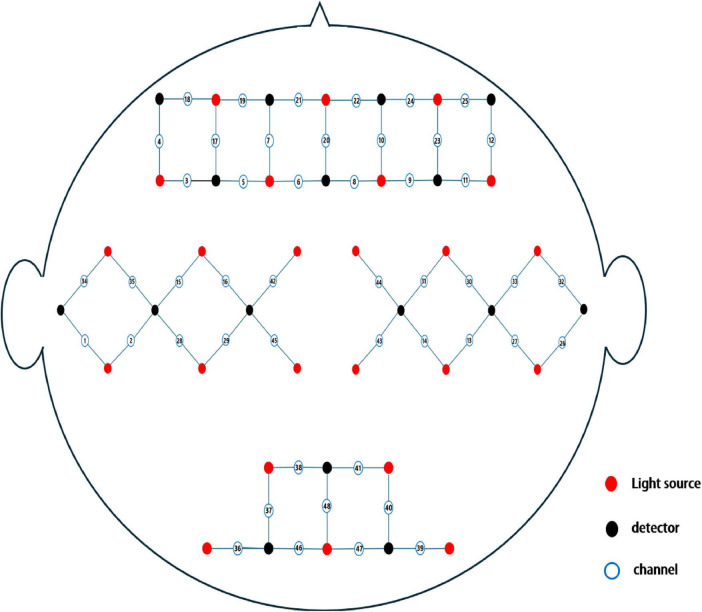
Detailed schematic of the functional near-infrared spectroscopy (fNIRS) layout.

The fNIRS signals were acquired using a multi-channel system (NirSmartII-3000A, Danyang Huichuan Medical Equipment Co., Ltd., China) with a sampling rate of 11 Hz and dual wavelengths (730 and 850 nm). Each participant wore a stretchable hood that covered the frontal, bilateral temporal, parietal, and occipital lobes. The cap comprised 21 sources and 16 detectors with spacings of 3 cm, resulting in 48 measurement channels. The spatial coordinates of the sources, detectors, and anchor points (at Nz, Cz, Al, Ar, and Iz, following the international 10–20 electrode placement system) were determined using an electromagnetic three-dimensional (3D) digitizer (Patriot, Polhemus, Colchester, Vermont, United States) on the head mold (Figure 3). Supplementary Table 1 presents the anatomical regions corresponding to each channel and their respective coverage percentages. The obtained coordinates were converted to the Montreal Neurological Institute (MNI) space and mapped onto the MNI standard brain template using the spatial registration method provided by NirSpace (China Limited, Danyang).

### Data analysis

The fNIRS signals were pre-processed using NirSpark V1.8.1 (Danyang Huichuang Medical Equipment Co., Ltd, Danyang). First, head motion was corrected. Second, digital band-pass filtering was applied for the range of 0.01–0.2 Hz. Third, the relative oxyhemoglobin, deoxyhemoglobin, and total hemoglobin concentration curves were obtained using the modified Beer–Lambert law (Wang et al., 2017). The path length factor for each wavelength was set to 6 to determine the relative deoxyhemoglobin, oxyhemoglobin, and total hemoglobin concentrations. Finally, the post-preprocessing channel integrity was verified, and missing data were excluded. By applying a band-pass filter (0.01–0.2 Hz) to each participant’s data during the video viewing, the ΔOD signals were converted into Δ[HbO2] and Δ[Hb]. The Pearson correlation coefficients between the time series of different channels were calculated to construct a functional connectivity correlation matrix (R-value) for each participant during the video viewing period. These R-values were subsequently transformed into Fisher’s z-scores to approximate a normal distribution for further statistical analysis to assess intergroup differences in functional connectivity (Homae et al., 2010; Wu et al., 2022).

### Statistical analysis

The data are presented as the mean ± standard deviation. Statistical analysis was conducted with R, and GraphPad Prism 8 (GraphPad Software, San Diego, CA, United States) was used to generate graphs. A paired *t*-test was used to determine differences in fNIRS data, facial expressions, and vital signs between the groups. A two-tailed *p*-value < 0.05 was deemed statistically significant.

## Results

### Demographics

Group A (low-frequency pornographic video watching) comprised 16 subjects with an average age of 19 years and included 12 men (75%) and four women (25%). Group B (high-frequency pornographic video watching) comprised five subjects with an average age of 19 years and included four men (75%) and one woman (25%). There were no significant differences between the groups in demographic variables (Table 1).

**TABLE 1:** Demographic information.

Category	Group A (*n* = 16)	Group B (*n* = 5)	t/χ ^2^	*P*
Age	21.27 ± 1.87	21.00 ± 1.00	t = 0.3057	0.767
Sex	Male: 12 (75%)	Male: 4 (75%)	χ^2^ = 0.0525	0.819
	Female: 4 (25%)	Male: 1 (25%)	–	–

### Questionnaire results

The means and standard deviations for each questionnaire are shown in Table 2. The high-frequency group (group B) had significantly higher PIPUS scores than the low-frequency group (group A). Additionally, there were significant differences in SDS and SAS scores between the groups, with group B scoring higher than group A.

**TABLE 2:** Questionnaire scores.

Variable (M ± SD)	Group A (*n* = 16)	Group B (*n* = 5)	*t*	*P*-value
PIPUS	12.5 ± 6.81	40.8 ± 10.68	–7.0940	0.000
SDS	32.5 ± 3.41	39.2 ± 6.81	–3.0046	0.0036
SAS	40 ± 3.22	53 ± 6.40	–6.1899	0.000

PIPUS, problematic Internet pornography use scale; SDS, self-rated depression scale; SAS, self-rated anxiety scale.

### Functional connectivity

**FIGURE 4:**
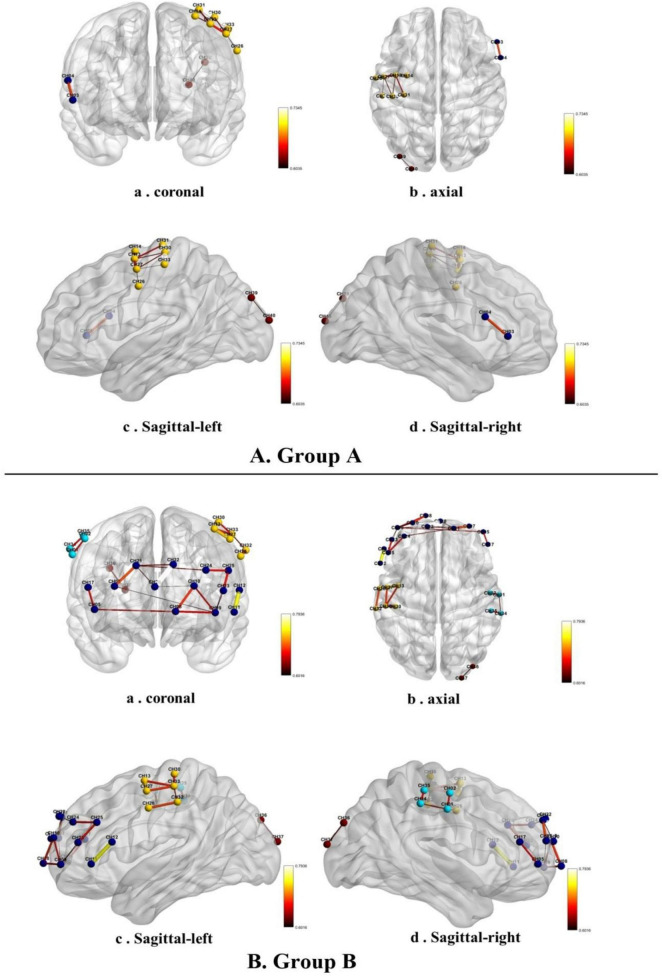
Functional connectivity in the low-frequency **(A,B)** high-frequency groups while they watched a pornographic video.

The fNIRS signals collected during the 10 min session of online pornography viewing were used to perform functional connectivity (FC) analyses without dividing the 10 min signal into several epochs for analysis. A custom MATLAB code was used to visualize the functional connectivities in groups A and B while they viewed the pornographic video. Group A presented significantly stronger functional connectivity in the inferior prefrontal cortex, pars triangularis of Broca’s area, the pre-motor cortex, supplementary motor cortex, the primary somatosensory cortex of the right parietal lobe, and the visual association cortex. From a coronal perspective, group B presented significantly stronger functional connectivity in the dorsolateral prefrontal cortex, frontopolar area, inferior frontal cortex, and pars triangularis of Broca’s area than group A (Figure 4A). From an axial perspective, group B presented significantly stronger functional connectivity in the primary somatosensory cortex, pre-motor cortex, and supplementary motor cortex in the parietal lobe (Figure 4B). From a sagittal left/right perspective, group B presented significantly stronger functional connectivity in the dorsolateral prefrontal cortex, inferior frontal cortex, the pars triangularis of Broca’s area in the prefrontal lobe, the primary somatosensory cortex, pre-motor cortex, and supplementary motor cortex in the parietal lobe (Figures 4, c, d). The threshold was set at 80% of the lesser maximum threshold from the two groups (Ren et al., 2022). Group A exhibited a lower maximum threshold than group B, with a value of 0.74. Therefore, the threshold for both sets of connections was set to 0.58.

The functional connectivities between the right pars triangularis of Broca’s area and frontopolar area, as well as between the right inferior prefrontal cortex and frontopolar area, were significantly different between groups A and B (Table 3). However, there were no significant differences between the two groups after correcting the false discovery rate.

**TABLE 3:** Differences in functional connectivity strength in the low-frequency (A) and high-frequency (B) groups while they watched pornography.

Region of interest	Group A mean	Group B mean	Group A standard deviation	Group B standard deviation	*t*	*P*
Right pars triangularis Broca’s area–frontopolar area	0.05937	0.346846	0.190101	0.344612	–2.38466	0.028303
Right inferior prefrontal gyrus–frontopolar area	0.152891	0.405062	0.169946	0.341663	–2.21957	0.039534

### SCWT

**FIGURE 5:**
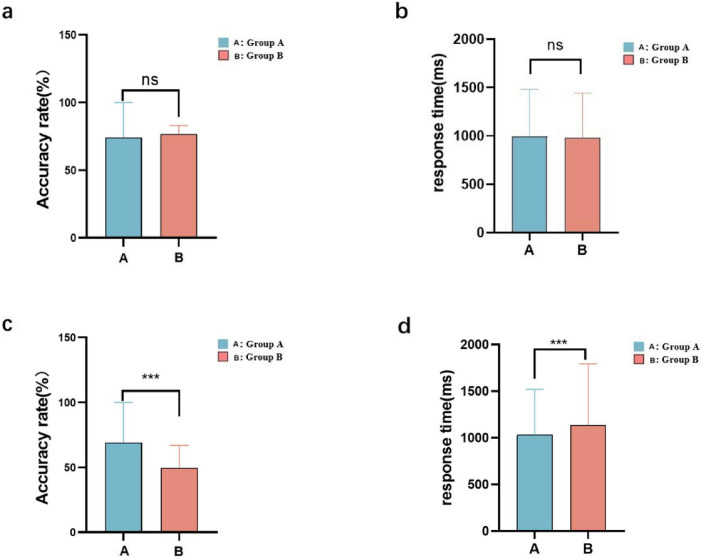
Stroop Color and Word Test (SCWT) results for the low-frequency **(A)** and high-frequency **(B)** groups: accuracy **(a,c)** and response time **(b,d)** before and after viewing the pornography (*** < 0.01, ns > 0.5).

The results of the SCWT were compared between groups A and B before and after viewing pornography. Before watching the pornographic video, there was no difference between the groups in response accuracy (Figure 5a). After watching the pornographic video, group A showed a significantly higher accuracy than group B (Figure 5c). The response time was not significantly different between the groups before watching the pornography (Figure 5b), but it was significantly shorter in group A after watching the pornography (Figure 5d).

### Life signs

**FIGURE 6:**
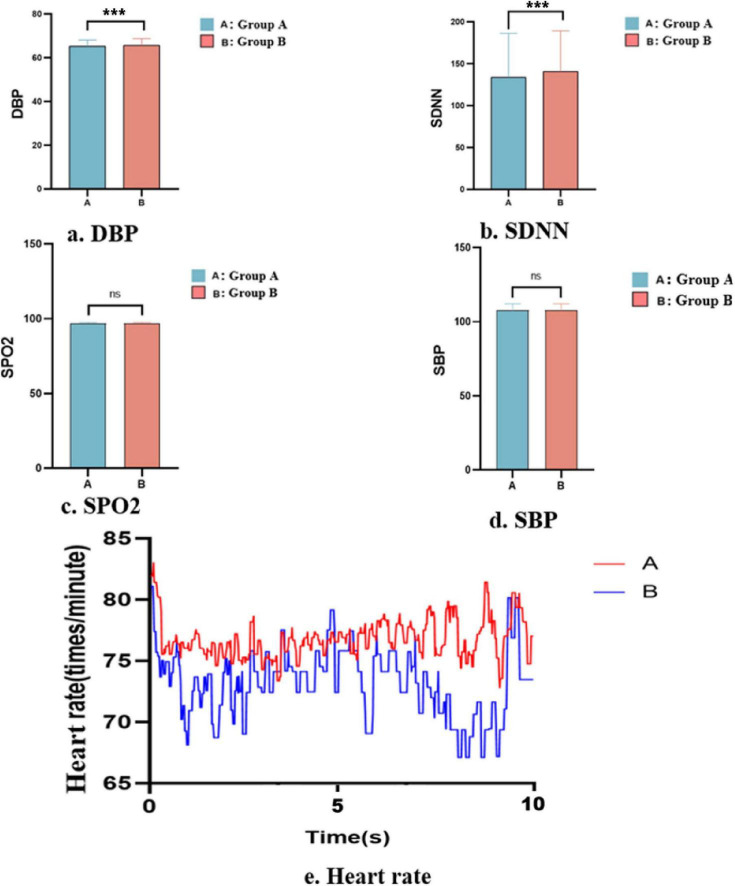
Life signs in the low-frequency (A) and high-frequency (B) groups: **(a)** diastolic blood pressure, **(b)** SD of normal-to-normal intervals, **(c)** percutaneous oxygen saturation, **(d)** systolic blood pressure, and **(e)** heart rate (*** < 0.01, ns > 0.5).

Life signs were recorded while the participants were watching the pornography. Compared with group B, group A presented a significantly higher DBP (*t* = –6.28, df = 10368, *p* < 0.0001, Figure 6a) and a significantly lower SDNN (*t* = –5.776, df = 10368, *p* < 0.0001, Figure 6b). No significant difference in SPO_2_ (Figure 6c) or SBP (Figure 6d) was found between groups A and B. Group A also showed a significantly higher heart rate (*t* = 17.4012, df = 10368, *p* < 0.0001, Figure 6e) than group B (Figure 6 and Supplementary Table 2).

### Facial expressions

**FIGURE 7:**
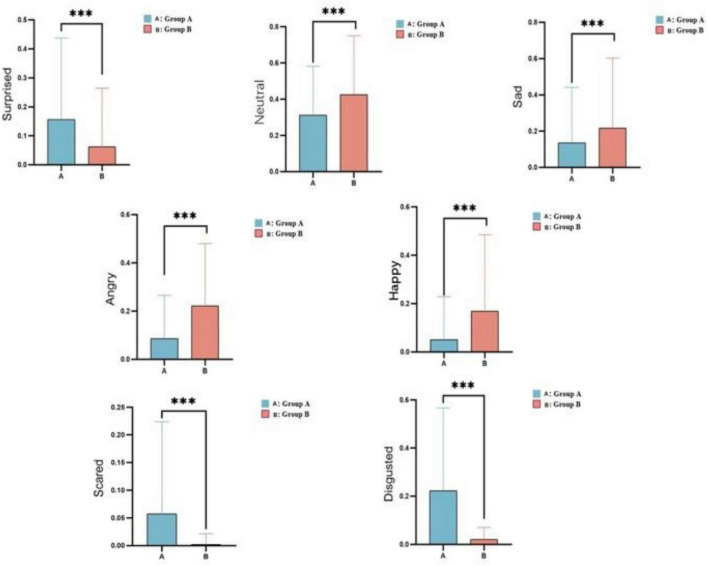
Facial expressions in the low-frequency (A) and high-frequency (B) groups while they watched pornography. The x-axis represents the groups, and the y-axis represents the mean values (*** < 0.01).

The facial expressions were clearly measured using the Face Reader software from Noldus Co., Ltd. While watching pornography, group B showed significantly stronger feelings of pleasure and higher levels of happiness than group A (*t* = –100, df = 231504, *p* < 0.0001). However, the expressions of anger (*t* = *-*101, df = 231504, *p* < 0.0001) and sadness (*t* = –45.6389, df = 231504, *p* < 0.0001) were significantly higher in group B, suggesting greater emotional fluctuations within this group. Additionally, the neutral or vacant expressions (*t* = –73.0421, df = 231504, *p* < 0.0001) were more dominant in group B, suggesting a deeper immersion in the pornography. Expressions of fear (*t* = 65.624, df = 231504, *p* < 0.0001), surprise (*t* = –73.0421, df = 231504, *p* < 0.0001), and disgust (*t* = 116.05, df = 231504, *p* < 0.0001) were significantly higher in group A than group B (Figure 7 and Supplementary Table 3).

## Discussion

There is a growing awareness that chronic consumption of pornography may lead to dependence, but the specific neurobiological mechanisms have not been deciphered (Tan et al., 2022). We found that the frequency of sexual arousal induced by pornography was significantly higher in the group that watched pornography more frequently (group B) than the group that watched pornography less frequently (group A). Compared with group A, group B exhibited enhanced functional connectivity in the primary sensory cortex and supplementary motor cortex. Group B participants exhibited higher parasympathetic nervous system activity than the group A participants, indicating that the video elicited a stronger sexual arousal in the high-frequency viewers. Volkow et al. (2003) indicated that enhanced sexual arousal drives the increased consumption of internet pornography.

While watching pornography, group A exhibited enhanced functional connectivity in the ventrolateral prefrontal cortex, which overlaps with the rewards system pathways. Group B presented significantly enhanced functional connectivity in the frontopolar region, ventrolateral prefrontal cortex, and dorsolateral prefrontal cortex. Similarly, drug abuse activates the midbrain dopamine (DA) pathway originating in the ventral tegmental area (VTA) and projecting to the nucleus accumbens. This midbrain–limbic dopamine pathway, known as the rewards center, connects with three key regions to form the so-called rewards system: the amygdala (positive and negative emotions and emotional memory), the hippocampus (processing and extracting long-term memory), and the prefrontal cortex (coordinating and deciding behavior), including the dorsolateral prefrontal and ventromedial prefrontal cortices (Zattoni et al., 2020). Importantly, we found that the functional connectivity in the prefrontal cortex with internet pornography addiction and drug addiction exhibits similar features (Gu et al., 2021). Moreover, the functional connectivity patterns in the brain’s cortex with internet pornography addiction are strikingly similar to those observed in schizophrenia.

In opioid drug users, drug consumption results in intense calmness, euphoria, analgesia, and a sense of blurred perception, thereby inhibiting the sympathetic nervous system and leading to a decreased heart rate and increased SDNN (Schwartz, 1998). We found that both groups showed a significantly reduced heart rate while watching the pornographic film, with group B showing a more pronounced decrease in heart rate and a larger SDNN. These physiological changes are similar to those observed during opioid drug use (Opioids, 2012). In addition, group B appeared more pleased than group A and also had more numb expressions, similar to the intense calmness, euphoria, analgesia, and blurred perception brought about by opioid drugs during consumption (Byrne, 1995).

The treatment for internet pornography addiction usually begins with psychotherapy, such as acceptance and commitment therapy. If uncontrolled, anti-androgens such as progesterone, which are considered to reduce libido, and serotonin-reuptake inhibitors, which can reduce sexual thoughts and compulsive behaviors, may be used (Sniewski et al., 2018). There is controversy about whether to use the opioid receptor antagonist naltrexone to treat internet pornography addiction (Sharma et al., 2022). Our findings suggest that being obsessed with viewing internet pornography has characteristics similar to opioid addiction.

We also found that internet pornography affects cognition and emotion. After watching the pornographic video, both groups showed a significant decrease in accuracy and an increase in reaction time in the SCWT, with group B showing a more pronounced decline. The fNIRS findings revealed that group B presented stronger functional connectivity between the inferior frontal cortex, which is associated with executive functions, inhibitory control, and emotion regulation, and the frontopolar region, involved in advanced cognitive functions such as decision-making, planning, and problem-solving (Cha et al., 2016; Li et al., 2020; Peña et al., 2022). Moderate enhancement of brain connectivity could contribute to improved brain function, but excessive increases in brain connectivity may be the reason for the observed short-term cognitive decline and emotional abnormalities. In long-term studies on internet pornography, researchers have found that negative emotions such as anxiety and depression are closely related to pornography consumption (Donadelli and Lalanne, 2020; Privara and Bob, 2023; Rolls et al., 2020). Our study also supports this viewpoint, with group B scoring significantly higher on the SAS and SDS questionnaires than group A. These results are consistent with the findings of prior studies (Prantner et al., 2024). By using fNIRS to scan brain functional connectivity under high-stress conditions, it was found that the connectivity between the left and right dorsolateral prefrontal cortex (DLPFC) and the left orbitofrontal cortex significantly increased (Al-Shargie et al., 2022), which is similar to the changes observed in brain connectivity in our high-frequency group. Some studies have suggested that internet pornography consumption may serve as a defense mechanism against excessive stress (Privara and Bob, 2023). Some studies have found that internet pornography is invisibly associated with compulsive sexual behavior, influencing cognitive processes while affecting emotions (Brand et al., 2016; Engel et al., 2024). At the same time, cognitive therapy has been shown to be effective in treating internet pornography addiction (Roza et al., 2024).

Although the experimental results showed significant differences in brain functional connectivity before FDR correction, facial expressions, life signs, and other measures, recruiting participants who consume internet pornography, particularly those who do so frequently, turned out to be a significant challenge. Our experiment was also restricted by ethical limitations, resulting in few high-frequency participants being recruited. This, in turn, amplified random errors and may have affected the experimental outcomes. Current fNIRS results post FDR failed to find significant difference, this may due to the frequency of porn video watching of recruited participants remains low.

## Conclusion

The effects of internet pornography addiction on brain functional connectivity in the prefrontal lobe exhibit characteristics similar to those of drug addiction. Moreover, individuals who frequently consume internet pornography report that they experience stronger sexual arousal and heightened pleasure while viewing, which subsequently adversely affects their cognition and emotions. Further research is needed to follow up on these preliminary findings.

## Data Availability

The original contributions presented in this study are included in the article/Supplementary material, further inquiries can be directed to the corresponding authors.
