# Correction: The impact of internet pornography addiction on brain function: a functional near-infrared spectroscopy study

**DOI:** 10.3389/fnhum.2025.1658882

**Published:** 2025-07-17

**Authors:** Qicheng Shu, Shiyu Tang, Zhenhua Wu, Jiahuan Feng, Wenhao Lv, Min Huang, Fan Xu

**Affiliations:** ^1^Department of Evidence-Based Medicine and Social Medicine, School of Public Health, Chengdu Medical College, Chengdu, China; ^2^Department of Clinic Medicine, School of Clinical Medicine, Chengdu Medical College, Chengdu, China; ^3^Department of Pharmacy, School of Pharmacy, Chengdu Medical College, Chengdu, China; ^4^Department of Physiology, School of Basic Medical Sciences, Chengdu Medical College, Chengdu, China

**Keywords:** internet pornography, addiction, functional near-infrared spectroscopy (fNIRS), brain function, cognition

In the published article, the reference: Levin, R. J. (2003). The ins and outs of vaginal lubrication. *Sexual Relationsh. Therapy* 18, 509–513. doi: 10.1080/14681990310001609859 appeared in the article. It has now been removed.

In the published article, there was an error. A correction has been made to **Discussion**, paragraph 1. This sentence previously stated: “Levin (2003) reported that enhanced functional connectivity in these brain regions is associated with sexual arousal, sexual activity, genital sensation, and autonomic movement of the genitals.”

This sentence has now been removed.

The original article has been updated.

