# Supplementary material for: The impact of internet pornography addiction on brain function: a functional near-infrared spectroscopy study
Source: Front Hum Neurosci. 2025 Apr 16;19:1477914. doi: 10.3389/fnhum.2025.1477914 (PMC12040873; doi:10.3389/fnhum.2025.1477914)
Supplement: Supplementary file 2 [file Table_2.DOCX]

# Supplementary File

**Table 2 The results of vital signs difference analysis**

| **Variable** | **Group** | **Mean** | **Std. dev** | **T** | **P** |
| --- | --- | --- | --- | --- | --- |
| Heart-rate | A | 76.862 | 9.298 | 17.4012 | <0.001 |
|  | B | 73.237 | 7.371 |  |  |
| Spo2 | A | 96.990 | 0.401 | -1.7933 | 0.073 |
|  | B | 97.007 | 0.409 |  |  |
| SDNN | A | 133.963 | 52.276 | -5.7760 | <0.001 |
|  | B | 140.920 | 48.445 |  |  |
| sbp | A | 107.680 | 4.424 | 1.2554 | 0.209 |
|  | B | 107.594 | 4.576 |  |  |
| dbp | A | 65.326 | 2.812 | -6.2809 | <0.001 |
|  | B | 65.747 | 3.004 |  |  |

**Table 3 The results of facial expression difference analysis**

| **Variable** | **Group** | **Mean** | **Std. dev** | **T** | **P** |
| --- | --- | --- | --- | --- | --- |
| Neutral | A | 0.312 | 0.270 | -73.0421 | <0.001 |
|  | B | 0.425 | 0.325 |  |  |
| Happy | A | 0.051 | 0.178 | -100 | <0.001 |
|  | B | 0.169 | 0.316 |  |  |
| Sad | A | 0.136 | 0.306 | -45.6389 | <0.001 |
|  | B | 0.217 | 0.385 |  |  |
| Angry | A | 0.086 | 0.180 | -101 | <0.001 |
|  | B | 0.222 | 0.258 |  |  |
| Surprised | A | 0.156 | 0.281 | 62.7252 | <0.001 |
|  | B | 0.062 | 0.203 |  |  |
| Scared | A | 0.057 | 0.167 | 65.6244 | <0.001 |
|  | B | 0.002 | 0.020 |  |  |
| Disgusted | A | 0.223 | 0.345 | 116.0488 | <0.001 |
|  | B | 0.021 | 0.049 |  |  |
